# NEWS

**DOI:** 10.7189/jogh.02.010201

**Published:** 2012-06

## AFRICA

• In Sierra Leone, traditional birth attendants (TBAs) were banned from assisting deliveries some 18 months ago, when the Sierra Leone government introduced their free health care initiative. Under this initiative, pregnant women receive support so long as they deliver in a clinic or hospital. The pros and cons of TBAs is the subject of fierce debate among health care professionals worldwide. WHO argues that, until there are sufficient midwives, the best policy is to train the TBAs in simple outreach work so that they can monitor low-risk pregnancies while referring more complicated cases to the clinics. (*The Guardian*, 17 Jan 2012)

• Medecins Sans Frontieres (MSF) has shut down two major medical centres in the Mogadishu, Somalia, after two of its aid workers were shot dead by a former colleague last month, the international medical aid agency reported in January (*Reuters*, 19 Jan 2012)

• Ivory Coast is abandoning free health care for all after a brief experiment, because the costs escalated rapidly, health minister Mr Yoman N'dri said in Abidjan. From February 2012, the free service will only be available to mothers and their children under six years of age. Aid organisations say the government move is understandable, given the country's recent political turmoil. (*The Guardian*, 27 Jan 2012)

• In April this year, The Ghana Health Service became the first African country to simultaneously introduce pneumococcal and rotavirus vaccines in its national immunization programme, in a bid to fight pneumonia and diarrhoeal diseases. The ceremony at which the vaccines were introduced also served as the platform for the official launch of the second African Vaccination Week (AVW), which is being observed from 23 to 28 April. Ghana is receiving backing from the GAVI Alliance. As well as being immunised against rotavirus and pneumococcal disease, children will also continue to be vaccinated against polio and yellow fever, as well as receiving the pentavalent vaccine (five in one) which protects against diphtheria, tetanus, whooping cough, hepatitis B and *Haemophilus influenza* type b. (*Ghana News Agency*, 26 Apr 2012)

• African economic growth in the past decade, averaging around 5% on the continent, is certainly good news compared with two decades of increasing poverty. But the reason for that growth is mainly the large-scale export of commodities, with no clear industrial or institutional benefits. “Jobless growth”, the source of the uprisings in north Africa, is the norm in Africa; although manufacturing exports quadrupled to over US$ 100 billion in the last decade, manufacturing is actually declining as a proportion of GDP from a fairly stable 17% (between 1965 and 1990) to 13% today. (*The Guardian*, 26 Apr 2012)

## ASIA

• After years of decline, with only 25 polio cases reported in Afghanistan in 2010, last year the number tripled to 76, according to the Afghan Ministry of Public Health. While the total remains small, polio is highly contagious and each detected case is likely an indicator of hundreds of ‘silent’ ones in children with mild infections, who become carriers. Health workers are alarmed at the reversal of trend, particularly since some of the cases erupted far outside the disease’s traditional areas in Afghanistan. (*New York Times*, 16 Jan 2012)

• Vietnam has asked international health experts to help investigate a mystery illness that has killed 19 people and sickened 171 others in an impoverished district in central Vietnam. The affected were mostly children and young people. The disease begins with a high fever, loss of appetite and a rash that covers the hands and feet, and it responds well to treatment if detected early, but re-infections are common. The disease was first detected in April 2011, but diminished by October last year, with a fresh epidemics wave starting in March 2012. (*Associated Press*, 20 Mar 2012)

• Indonesia is now the world's fifth pentavalent vaccine producer, after the state pharmaceutical firm PT Bio Farma has successfully developed it to meet domestic and foreign needs. The company would also sell the vaccine abroad after securing a certificate from the World Health Organization (WHO), while an estimated 4.8 million infants in Indonesia would receive this vaccine. (*Antara News*, 24 Mar 2012)

• The Pakistani medical official who ran a fake CIA vaccination programme to help find Osama bin Laden has been jailed for 33 years. Dr Shakil Afridi may now face decades in jail, despite calls from senior US officials to release the man who helped to track down the al-Qaida chief. (*The Guardian*, 23 May 2012)

• Japan’s Senior Vice Minister of Finance, Yukihisa Fujita, announced at the African Development Bank’s (AfDB) Annual Meetings in June 2012 that the Japanese government intends to provide another US$ 1 billion over the next five years for a second phase of the Enhanced Private Sector Assistance (EPSA) for Africa Initiative, as a follow-up to the G8 Camp David Summit which took place from 18-19 May 2012. (*AfDB*, 02 Jun 2012)

## AUSTRALIA AND WESTERN PACIFIC

• The World Health Organization warned that the battle against the age-old scourge of leprosy is not yet over, with more than 5000 new cases reported yearly in the Western Pacific, where the disease was declared eliminated in 1991. (*Washington Post*, 13 Feb 2012)

• Australia-based advocate Dr Kate Armstrong voiced her concern in March 2012 that children’s needs - let alone their rights - are still being forgotten. In her analysis, the targets now being considered by the World Health Organization and others to reduce the impact of heart disease, cancer and other non-infectious diseases are in danger of being focused solely on adults. Children in the poorest countries die of cancer and asthma and diabetes, but the targets under consideration aim to bring down the deaths of adults over the age of 30. (*The Guardian*, 19 Mar 2012)

• Speaking at the Australian National University in Canberra, GAVI CEO Dr Seth Berkley delivered an in-depth speech outlining the crucial role Australian funding will play in saving lives in the developing world over the next decade. He explained that the unique financial structure of GAVI had allowed it to drive a 97% reduction in the cost of the pneumococcal vaccine, driving strong outcomes in the world's 73 poorest nations. Dr Berkley also touched on the need to drive access to HPV vaccine, created by Australian scientist, Professor Ian Frazer. (*GAVI Alliance*, 21 March 2012)

• Australia has released a review set to inform the country’s future funding and engagement with multilateral organizations and development banks. The Australian Multilateral Assessment measures the effectiveness of Australia’s key multilateral partners against a number of components, such as alignment with Australia’s aid priorities and interests, cost and value consciousness, and transparency and accountability. (*Devex*, 02 Apr 2012)

• Australian researchers are preparing to expand trials for malaria vaccines as drug-resistant strains emerge in developing countries. Swiss non-profit Medecins for Malaria has given A$ 500 000 to the Queensland Institute of Medical Research (QIMR) to expand its trials of malaria vaccines. (*Australian Associated Press*, 26 Apr 2012)

## CHINA

• The Chinese drug artemisinin has been hailed as one of the greatest advances in fighting malaria since the discovery of quinine centuries ago. Artemisinin’s discovery is being talked about as a candidate for a Nobel Prize in Medicine. In one of the paradoxes of history, the drug was discovered thanks to Mao Zedong, who was acting to help the North Vietnamese in their jungle war against the Americans, after which it 'disappeared' for 30 years because of China’s isolation and the indifference of Western donors, health agencies and drug companies. (*New York Times*, 16 Jan 2012)

• According to the newspaper affiliated to China's health ministry, one year on from the World Health Organization (WHO) freeing Chinese vaccine producers to apply for rights to distribute their products globally, none have qualified to do so. At present, only the Henan-based Hualan Biological Bacterin Co. Ltd, and the Chengdu subsidiary of China National Biotec Group have submitted applications for this “WHO license” for their seasonal flu vaccine and Japanese encephalitis vaccine, respectively. They have not yet won approval. China has 36 of the world's 85 vaccine producers, but without the WHO approval the only route for Chinese vaccines to be distributed globally is if they are donated, or if foreign countries are approached individually. (*Xinhua*, 29 Feb 2012)

• China hopes to cap the number of people living with HIV/AIDS at 1.2 million by 2015, up from around 780 000 at present, partly by promoting increased condom use. While praising achievements made over the past few years, including improved life expectancy for AIDS patients, the State Council said that China still faced a difficult task to prevent the spread of the disease. (*Reuters*, 29 Feb 2012)

• Tracking the prevalence of the diseases of affluence, the World Health Organization (WHO) reported that a quarter of those 25 or older now have high blood pressure worldwide, and almost one in 10 has worrying levels of glucose in their blood. The WHO’s tally of the latest global health statistics for the first time includes a look at blood pressure and glucose levels, two of the risk factors for diabetes and cardiovascular disease. China will likely be the most significant example among the low and middle-income countries expected to develop strategies and health policies to tackle this problem on a large scale. (*Washington Post*, 16 May 2012)

• At the start of the 65th World Health Assembly, WHO Chief Dr Margaret Chan said that in 2011, “…after extensive technical collaboration, WHO prequalified China's State Food and Drug Administration”. She highlighted China's potential for increased vaccine production at lower prices following WHO approval of national vaccine regulator. Effective regulatory oversight is essential since vaccines are used on a population wide basis and are usually given to healthy infants. (*AllAfrica*, 21 May 2012)

## EUROPE

• Gordon Brown is making a call for the international community to make education a higher priority and to develop a plan to achieve universal primary education by 2015. The former UK prime minister wants to create a “global fund for education”, which would raise the Ł13bn per year needed to bring lessons to the poorest children. (*BBC News*, 25 Jan 2012)

• The French cabinet agreed to pursue a tax on financial transactions that they hope will eventually be adopted by other European countries. The project will see a 0.1% tax on buying shares belonging to firms with a French headquarters and more than one billion euros in capital. The French finance ministry estimates the tax, which if passed by parliament would take effect on August 1, will bring in € 1.1 billion (US$ 1.45 billion) annually. (*AFP*, 08 Feb 2012)

• In February, Norway announced a new initiative aimed at addressing gaps in the research on the importance of investment in female health as a particularly strong driver of sustainable economic development. This work will be undertaken under the auspices of a network of global leaders chaired by the Norwegian Foreign Minister Jonas Gahr Store and include representatives from key agencies, Bill and Melinda Gates Foundation, The Lancet, WHO and World Bank. (*NORAD*, 14 Feb 2012)

• Brian Greenwood has been named the winner of the Canadian Gairdner Foundation's 2012 Global Health Award for his “contributions to significantly reducing mortality in children due to meningitis and acute respiratory infection and for contributions to malaria prevention”. His laboratory did the pivotal epidemiological studies that showed the importance of pneumonia and meningitis as major causes of death in young African children, a fact not widely appreciated at that time. His team consequently set up a series of trials of vaccines against *Haemophilus influenzae* type b (Hib) and *Streptococcus pneumonia* – the most frequent bacterial causes of pneumonia and meningitis in young African children. The success of two of these trials contributed to the decision by WHO to recommend immunisation with Hib and pneumococcal conjugate vaccines in countries with high child mortality. (*The Lancet*, 26 Mar 2012)

• A global initiative, designed to strengthen research into non-communicable diseases and improve collaboration between rich and poorer countries, has been launched in London. The Centre for Global Non-Communicable Diseases at the London School of Hygiene and Tropical Medicine aims to foster new studies and ensure research evidence is acted upon by policymakers. (*BMJ*, 25 Apr 2012)

## INDIA

• The Prime Minister Singh said that India will press science and technology into serving a national policy of more inclusive, sustainable and rapid growth for its people. Singh underscored the need to use innovations creatively for social benefit, and “give practical meaning to innovation””. (*The Guardian*, 4 January 2012)

• India's pioneering “Home-based New Born Scheme” has shown the world a new way to cut down on neonatal mortality, which occurs within 28 days of birth. Almost 13 years after Dr Abhay Bang demonstrated a 62% reduction in neonatal mortality through multiple home visits in Maharashtra, the World Health Organization (WHO) has touted it as a global policy. (*Times of India*, 16 Feb 2012)

• More people in India own a mobile phone than a toilet at home, according to the latest census data. Nearly half of India's 1.2 billion people have no toilet at home; only 46.9% of the 246.6 million households have lavatories, while 49.8% defecate in the open and the remaining 3.2% use public toilets. (*BBC News*, 14 Mar 2012)

• Bill Gates and Uttar Pradesh’s Chief Minister Akhilesh Yadav held a meeting in the state capital to define ways to improve the health and agriculture sector with the help of the Bill and Melinda Gates Foundation. It was decided that a memorandum would be signed between the state government and the Foundation, under which the Foundation would provide technical, management and program design support in the fields of maternal, neonatal, child health, vaccination and various other health and agriculture related areas. (*Economic Times*, 30 May 2012)

• The number of people with cancer is set to surge by more than 75% across the world by 2030, with particularly sharp rises in countries such as India, as they adopt unhealthy ‘Westernised’ lifestyles. A study published in *Lancet Oncology* predicted that middle-income countries could see an increase of 78% in the number of cancer cases by 2030. Cases in less developed regions were expected to see a 93% rise over the same period. Those rises would more than offset signs of a decline in cervical, stomach and other kinds of cancer in wealthier nations. (*Reuters*, 31 May 2012)

## THE AMERICAS

• The definition of autism is being reassessed by an expert panel appointed by the American Psychiatric Association. Proposed changes in the definition of autism would sharply reduce the surging rate at which the disorder is diagnosed now. However, it may make it harder for many people who would no longer meet the criteria to get health, educational and social services. For years, many experts have been suggesting that the vagueness of the current criteria for autism and related disorders was contributing to the ballooning rate of 1% children being affected, according to some estimates (*New York Times*, 19 Jan 2012)

• Gilead wants to be able to market Truvada, which is currently used as a HIV treatment, as a preventative pill to uninfected individuals. If approved, it would be the first of its kind. But the move has sparked debate among public health advocates who argue that the wide availability of the drug would discourage safe sex and would, in fact, increase the incidence of HIV. (*The Daily Mail*, 31 Jan 2012)

• The US President's budget request for 2013 proposed to cut the total money spent on global health initiatives by 3.5%, while PEPFAR's budget shrinks by 10.8%. However, contributions to the struggling Global Fund for AIDS, TB and Malaria should go up by 27%, to US$ 1.65 billion. In addition to the Global Fund, GAVI, IDA, Asian Development Fund and the African Development Fund are all among agencies that are getting increases on their funding; notably, GAVI by 11.5%. (*Center for Global Development*, 15 February 2012)

• Chagas disease, caused by parasites transmitted to humans by blood-sucking insects, has been named “the new AIDS of the Americas” in an editorial published in *PLoS Neglected Tropical Diseases*. The authors argue that the dangerous spread of Chagas through this hemisphere somewhat resembles the early spread of HIV. Chagas is also known as American trypanosomiasis, because the bugs carry single-celled parasites called trypanosomes. Like AIDS, Chagas disease has a long incubation time and is hard or impossible to cure. Chagas infects up to eight million people in the hemisphere, mostly in Bolivia, Mexico, Colombia and Central America; however, more than 300 000 of the infected live in the United States, many of them immigrants. (*New York Times*, 28 May 2012)

• Dr William Foege, who has been widely regarded as the health innovator behind the eradication of smallpox, has left an indelible mark in the field of global health. Because of his dedication and service to the public’s health, President Barack Obama today presented him with the Presidential Medal of Freedom, the nation’s highest civilian honour. (*Public Health Newswire*, 29 May 2012)

